# MERItest^TM^—A multidimensional model for functional evaluation at 6 months after ACL reconstruction

**DOI:** 10.1002/jeo2.70836

**Published:** 2026-07-30

**Authors:** Xavier Laurent, Damien Dodelin, Nicolas Graveleau, Nicolas Bouguennec

**Affiliations:** ^1^ Restart, Medical Stadium Mérignac France; ^2^ Biomechanical Laboratory, Medical Stadium Mérignac France; ^3^ Clinique du Sport Mérignac France

**Keywords:** ACL reconstruction, biomechanics, functional testing, movement quality, return to sport

## Abstract

At 6 months following anterior cruciate ligament reconstruction (ACLR), athletes transition from structured rehabilitation toward sport‐specific reconditioning, a critical phase characterised by elevated re‐injury risk and high variability in recovery trajectories. Yet clinical assessments at this milestone often rely on unidimensional metrics such as hop distance or limb symmetry indices (LSI), which fail to capture the complexity of functional readiness. This gap between apparent recovery and underlying neuromechanical deficits may contribute to persistently high re‐injury rates, even in athletes who meet conventional return to sport criteria. The MERItest^TM^ is presented as a structured, multidimensional evaluation framework designed to enhance clinical reasoning at the 6‐month follow‐up. Rather than introducing new isolated tests, it integrates three complementary domains: monoarticular isometric strength testing, three‐dimensional biomechanical analysis of functional tasks (jumping, hopping and running), and psychological readiness assessment using the anterior cruciate ligament‐return to sport after injury (ACL‐RSI) questionnaire, into a unified interpretive model. The isometric component quantifies contractile capacity of key muscle groups, the three‐dimensional biomechanical analysis captures movement strategies and intersegmental coordination under dynamic loading, and the psychological assessment contextualises motor behaviour through confidence and risk appraisal. By examining not only how much force an athlete produces, but how movement is organised and experienced, the framework enables identification of residual deficits and compensatory strategies often overlooked by performance‐based testing alone. The MERItest^TM^ proposes a shift from checklist‐driven clearance decisions toward multidimensional profiling and individualised interpretation of recovery patterns, thereby supporting more informed clinical reasoning. Its design balances biomechanical rigour with clinical feasibility, supporting integration in both research and specialised clinical environments. Although prospective validation is required to confirm its predictive utility, this framework reframes the 6‐month evaluation as a diagnostic opportunity rather than a binary clearance checkpoint.

**Level of Evidence:**

Level V.

**Abbreviations**
ACL: anterior cruciate ligament
ACLR: anterior cruciate ligament reconstruction
ACL‐RSI: anterior cruciate ligament‐return to sport after injury
CMJ: countermovement jump
LSI: Limb Symmetry Index
SLHD: single‐leg hop for distance

## INTRODUCTION

Anterior cruciate ligament (ACL) injuries are among the most prevalent and impactful injuries in sports medicine, particularly in young, athletic populations. Despite surgical reconstruction and rehabilitation, re‐injury remains alarmingly common: up to 25% of athletes aged 15–26 suffer a second ACL rupture [10]. This recurrence rate highlights a persistent blind spot in how functional recovery is assessed and managed. In most team sports involving cutting, jumping, or pivoting, ACL injuries occur predominantly through non‐contact mechanisms accounting for 84% in handball, 90% in volleyball or 47% in football [27]. These injuries often arise during offensive actions such as deceleration, change of direction, or single‐leg landing, which place high demands on neuromuscular control and intersegmental coordination. This pattern highlights that injury risk is less about contact forces and more about how the athlete moves and absorbs force under functional stress. Assessing movement quality is therefore essential to guide return to sport decisions and reduce re‐injury risk.

While a range of return to sport batteries and objective criteria have been proposed in the literature, most remain grounded in quantitative performance outputs such as hop distance, peak torque production, or limb symmetry indices. These measures are accessible and reproducible, yet they often fail to detect subclinical deficits, compensatory strategies, or movement adaptations that may increase reinjury risk [17]. Furthermore, the application of such tests is frequently reduced to a checklist logic, lacking integration into a broader clinical reasoning process. The result is a gap between what is measured and what is needed to support safe and effective rehabilitation and return to play pathways. Recent biomechanical frameworks have also emphasised the importance of clinically interpretable movement strategies and force‐time characteristics during high‐demand tasks, supporting a broader evolution toward integrated movement analysis in sports medicine [5]. Recent ACL rehabilitation literature has increasingly emphasised the need for more individualised and multidimensional rehabilitation approaches integrating physical, neurocognitive and psychological dimensions beyond isolated performance metrics and time‐based progression models [23].

In response to these limitations, some clinicians and researchers have developed localised, often innovative protocols that combine biomechanical testing, strength assessment and subjective measures. However, these initiatives remain highly heterogeneous, rarely standardised, and infrequently translated into reproducible clinical frameworks. Despite the accumulation of tests and metrics, there is no widely adopted, conceptually unified model that allows for the integration of physical, biomechanical and psychological dimensions of recovery at the mid‐term stage post‐anterior cruciate ligament reconstruction (ACLR). To address the limitations of conventional mid‐term evaluations post‐ACLR, the MERItest^TM^, a structured multidimensional assessment protocol, was developed to inform clinical reasoning at the 6‐month follow‐up. It integrates three complementary components: isometric strength testing, three‐dimensional biomechanical task analysis and psychological readiness evaluation. The isometric tests are performed in standardised monoarticular positions to quantify the maximal force‐generating capacity of key muscle groups under isolated and fatigue‐minimised conditions. While they do not replicate task dynamics, their simplicity and reliability make them well suited to detect residual strength deficits that may constrain motor control or joint loading strategies. In contrast, the three‐dimensional biomechanical component captures intersegmental coordination during functional tasks such as jumping, hopping and running. By analysing both kinetic and kinematic variables across joints and planes, it reveals how athletes organise and sequence movement particularly during tasks involving vertical and horizontal loading demands. Finally, the anterior cruciate ligament‐return to sport after injury (ACL‐RSI) questionnaire anchors the assessment in the psychological domain, recognising that confidence, fear and perceived readiness are tightly linked to neuromuscular behaviour and return to sport decisions.

Importantly, the novelty of the MERItest^TM^ does not reside in the individual components themselves, as isometric strength testing, three‐dimensional biomechanical analysis and psychological assessment are already well established in the literature. Rather, its originality lies in the structured organisation and interpretation of these domains within a unified clinical reasoning framework. The MERItest^TM^ is not intended as an additional test battery, but as an interpretive model designed to identify interactions and potential discordances between physical capacity, movement behaviour and psychological readiness. This narrative review outlines the scientific rationale, design principles and structure of the proposed framework within the broader context of post‐ACLR functional evaluation.

## RATIONALE FOR A MULTIDIMENSIONAL ASSESSMENT AFTER ACLR

The mid‐term phase following ACLR, particularly the 4–8‐month window, represents a critical transition from supervised rehabilitation to sport‐specific reconditioning. Clinical decisions made during this period have long‐term implications for re‐injury risk, tissue tolerance and athletic performance. Yet in many cases, clearance decisions are still based on fragmented, unidimensional metrics, typically limb symmetry index or basic hop distances, without deeper consideration of the neuromechanical strategies underpinning performance. At 6 months following ACLR the majority of patients have not recovered knee function across strength and performance domains. In a cohort of over 4000 patients, only 19.6% met limb symmetry thresholds on quadriceps strength, hamstring strength and single‐leg hop tests combined [3]. Moreover, the choice of thresholds is often arbitrary. These findings reinforce that 6 months post‐ACLR is not a 'clearance' stage, but rather a diagnostic window, a critical opportunity to identify which neuromuscular deficits remain. Movement efficiency, physical competency, intersegmental coordination and psychological readiness often remain impaired despite standard timelines. A multidimensional evaluation at this stage is essential not only to characterise recovery patterns, but also to inform individualised rehabilitation priorities, many of which may be missed by surface‐level or single‐domain testing.

Traditionally, return to sport criteria have emphasised quantitative performance metrics such as single‐leg hop distance, isokinetic strength, or limb symmetry indices. While these benchmarks offer reproducible reference points, they often fail to capture how the athlete achieves them [26]. Accumulating performance scores may give the illusion of readiness, yet it offers no assurance regarding the quality of movement or neuromuscular control. Recovery is not just defined by how far one can jump, but how well one lands as well. Many athletes who 'pass' objective tests continue to rely on compensatory motor strategies, such as load redistribution, reduced knee excursion, increased trunk or pelvic motion, prolonged ground‐contact times, or altered kinetic sequencing during landing and deceleration tasks. These patterns are consistently associated with a higher re‐injury risk [12, 15, 16, 25]. Without examining how movement is executed, clinicians risk overestimating function and underestimating vulnerability.

Beyond physical execution, psychological readiness has emerged as a determinant of both performance and safety. Fear of re‑injury, reduced confidence and altered risk appraisal influence motor behaviour, often leading to constrained movement patterns or protective strategies that affect loading symmetry and timing [7, 20, 29]. Incorporating validated measures such as the ACL‐RSI scale provides essential context to interpret physical data and align rehabilitation with the athlete's perceived readiness.

Taken together, these insights reinforce that functional recovery cannot be captured by performance metrics alone. Strength, movement quality and psychological state interact continuously to shape neuromuscular expression and movement safety. A multidimensional assessment at this stage allows clinicians to objectively profile recovery and individualise rehabilitation priorities, thereby bridging the persistent gap between apparent performance and readiness.

### CONCEPTUAL FOUNDATIONS OF THE MERITEST^TM^

The MERItest^TM^ protocol was developed in response to the recognised limitations of traditional post‐ACLR evaluations to adequately characterise recovery and the need for a clinically usable, yet biomechanically rigorous, assessment model [4, 21]. Rather than proposing an additional battery of isolated tests, the present framework offers a structured approach that aims to enhance clinical reasoning and enable individualised profiling of athletes at the 6‐month postoperative milestone.

### Diagnostic orientation

One of the primary goals of the framework is to move beyond binary 'clear/not clear' classifications and instead identify residual neuromuscular and biomechanical deficits that may remain concealed within conventional performance metrics. By combining isolated strength assessment with movement analysis during dynamic functional tasks, the protocol enables clinicians to identify *how* an athlete performs, not just *how much* they achieve. This diagnostic dimension is essential to distinguish between restored physical capacity and altered movement expression.

### Identification of clinically relevant movement patterns

The testing protocol also aims to support the identification of clinically relevant movement patterns. Through combined kinetic and kinematic analysis, it enables characterisation of compensatory strategies involving trunk, pelvic and lower‐limb coordination, especially during landing, propulsion and load absorption phases. This multidimensional interpretation may help reveal movement behaviours potentially associated with persistent vulnerability despite satisfactory global performance outcomes.

### Guidance for rehabilitation

Beyond assessment itself, the framework provides actionable data to support the progressive return to play process. The integration of isometric strength profiles with high‐resolution three‐dimensional motion analysis and psychological readiness allows clinicians to orient rehabilitation toward the specific mechanisms limiting recovery in each athlete, whether related to force production, dynamic control, load distribution, or psychological readiness.

### Practical and technological considerations

Although the MERItest^TM^ incorporates advanced biomechanical instrumentation, its structure was deliberately optimised to balance analytical depth and clinical feasibility. The protocol was restricted to a limited number of functional tasks selected for their biomechanical relevance and feasibility at the 6‐month stage post‐ACLR. Standardised isometric protocols and structured interpretation principles were integrated to facilitate reproducibility and interdisciplinary clinical use.

Overall, the MERItest^TM^ was conceived as a multidimensional interpretive model intended to bridge high‐resolution biomechanical assessment with individualised rehabilitation decision‐making during a critical stage of post‐ACLR recovery.

## DESCRIPTION OF THE MERITEST^TM^ PROTOCOL

**Figure 1:**
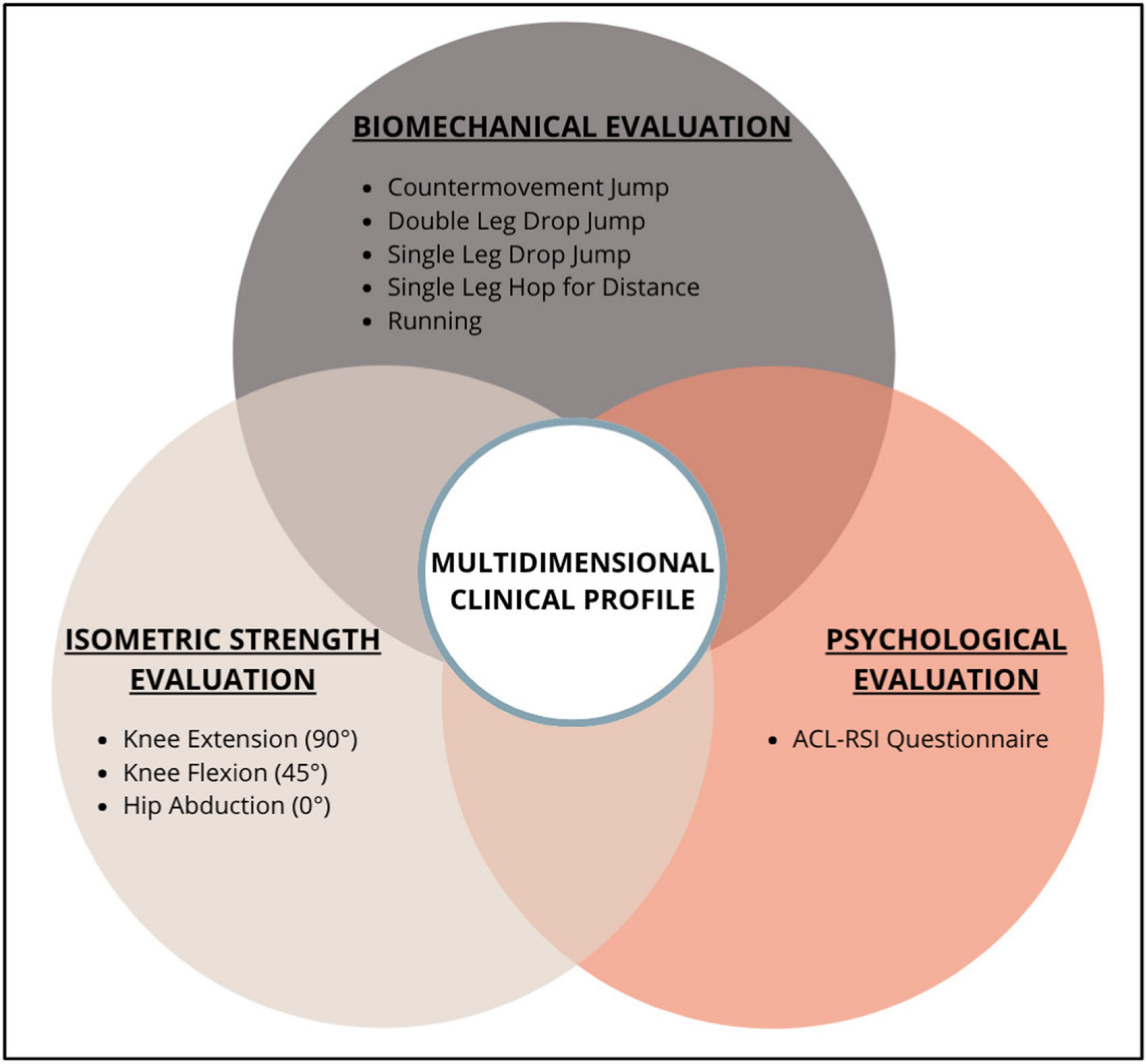
Conceptual diagram of the MERItest^TM^ protocol.

The present assessment framework integrates three primary components: isometric strength testing, three‐dimensional biomechanical analysis and psychological readiness evaluation to provide a multidimensional assessment of functional recovery six months after ACLR as presented in Figure 1. Each component was selected based on its clinical relevance, feasibility and potential to reveal residual deficits associated with reinjury risk or suboptimal athletic recovery.

### Isometric strength assessment

The MERItest^TM^ protocol includes a targeted isometric strength assessment aimed at quantifying unilateral peak force in key lower limb muscle groups: knee extensors (tested at 90° of knee flexion), knee flexors (prone at 30°) and hip abductors (side‐lying in neutral alignment). Testing is performed using a fixed‐frame dynamometer (VALD ForceFrame®), bilateral outputs are normalised and integrated to evaluate strength capacities. Unlike isokinetic evaluations, which involve coordinated joint movement and neuromotor synchronisation, isometric testing isolates maximum voluntary torque production at a fixed joint position [8]. This setup removes the influence of compensatory strategies or movement coordination, allowing clinicians to objectively assess the contractile capacity of individual muscle groups. It represents a foundational measure highlighting whether the athlete possesses sufficient neuromuscular resources before assessing how these are deployed during dynamic tasks such as landing. The rationale for selecting 90° of knee flexion in the knee extension test stems from evidence that torque deficits post‐ACLR often persist in this range. Authors showed that interlimb differences between 47° and 97° were still present up to 8 months after surgery, despite apparent recovery of peak torque values [14]. Additionally, within the 90°–110° range, the quadriceps displays optimal neuromuscular efficiency, generating high torque relative to EMG activation [18]. Importantly, static isometric outputs have also shown functional correlates. In a recent study, authors demonstrated that isometric quadriceps strength symmetry at 90° was more strongly associated with peak knee extension moments during countermovement jumping than isokinetic values, reinforcing its mechanical relevance for high‐force tasks [2]. Clinically, isometric testing offers additional advantages: it minimises fatigue, requires minimal setup and demonstrates high reliability [1]. Moreover, its monoarticular, non‐integrative nature stands in contrast to dynamic tasks that challenge multisegmental coordination. This dichotomy is deliberate within the present testing protocol: strength is initially captured independently from dynamic control, allowing clinicians to distinguish between true contractile deficits and movement neuromuscular issues observed during functional tasks. Recent evidence highlights that the temporal characteristics of force production play a key role in dynamic knee joint loading. In particular, explosive quadriceps capacity has been associated with landing mechanics following ACLR [11]. In this context, dynamic tasks such as jump and landing assessments may provide functional insight into these time‐dependent neuromuscular demands, complementing maximal isometric strength evaluation. Ultimately, isometric testing in the MERItest^TM^ is not a standalone benchmark, but an essential component within a multidimensional framework.

### Biomechanical assessment

A central element of the MERItest^TM^ protocol is the use of three‐dimensional motion analysis combined with synchronised ground reaction force data, obtained via a multicamera motion capture system (Qualisys) and force plates (AMTI). This integration allows for detailed analysis of kinematic and kinetic parameters during a selection of functional tasks chosen for their biomechanical relevance to post‐ACLR recovery.

The test battery includes the following tasks: bilateral countermovement jump (CMJ), bilateral and unilateral drop jump, single‐leg hop for distance (SLHD), and running on an instrumented walkway at auto‐selected moderate speed (10–12 km/h). Data acquisition focuses on both kinematic variables (joint angles, segmental displacements, inter‐limb coordination, frontal‐plane asymmetries, pelvic and trunk behaviour) and kinetic variables (vertical, horizontal and lateral ground reaction forces, joint moments) [19]. A detailed description of all biomechanical variables included in the analysis is provided in the supplementary file. This dual analysis provides insights not only into movement outcomes, but also into underlying control strategies. Drop jump tasks are particularly useful to assess lower limb stiffness, reactive force modulation and frontal‐plane trunk and pelvis control during impact absorption [13]. The SLHD emphasises propulsion and landing control under high unilateral loading. Meanwhile, the running task, performed on the instrumented track, enables the observation of continuous gait dynamics such as pelvic stability, loading strategies, sagittal joint excursions and coordination between trunk, pelvis and lower limbs. Rather than relying on binary thresholds or simplistic limb symmetry indices, the proposed evaluation protocol emphasises multivariate interpretation, with a focus on movement coherence, segmental alignment and compensatory patterns. The inclusion of both vertical and horizontal components, as well as segmental coupling, allows for a nuanced evaluation of movement quality and functional integration, two key dimensions in understanding residual deficits at mid‐term follow‐up.

### Psychological readiness evaluation

The third component of the MERItest^TM^ involves the assessment of psychological readiness to return to sport using the ACL‐RSI questionnaire [28]. This validated tool explores three psychological domains: emotional response, confidence in performance and risk appraisal. It generates a global score between 0 and 100, where lower scores reflect higher psychological inhibition and decreased perceived readiness. Psychological readiness, especially fear of reinjury, is important for return to sport readiness and has been shown to influence both neuromuscular control and return to sport decisions [22, 29]. For example, athletes with high levels of fear or low confidence may unconsciously adopt altered movement strategies that reduce mechanical loading but increase asymmetry or coordination deficits [24]. Conversely, those with excessive confidence and poor movement control may be at higher risk for early re‐injury. In the testing protocol, the ACL‐RSI score contributes to the overall profile and supports the clinical decision‐making process. A low score may prompt additional psychological support or training adaptation, even in the presence of satisfactory biomechanical and strength data. Conversely, discrepancies between physical and psychological indicators may highlight the need for further individualised monitoring. Recent evidence further suggests that psychological readiness and functional recovery may not evolve uniformly across individuals, with lower ACL‐RSI scores and delayed recovery patterns observed in certain subgroups, particularly among female athletes [9].

## CLINICAL INTEGRATION AND INTERPRETATION

The present testing framework was designed not merely to generate isolated test results, but to support integrated clinical reasoning. Each component—isometric, biomechanical and psychological evaluations—offers complementary information. When interpreted in combination, these data points enable the clinician to move from binary clearance decisions to a more nuanced understanding of the athlete's readiness profile. This interpretive framework relies on multidimensional profiling rather than threshold‐based algorithms. Rather than asking whether a limb symmetry index exceeds 90%, the proposed multidimensional approach encourages clinicians to ask: *Which neuromechanical strategies does this athlete rely on? Are these strategies robust, symmetrical, and transferable to sport‐specific tasks? Are they supported by sufficient strength and psychological confidence?*

### Profiling logic

Each athlete is interpreted across three complementary dimensions:

1. Force generation capacity—Identifying persistent deficits in isolated muscle groups (such as hamstring weakness, low hip abduction strength) that may influence load tolerance and movement control.
2. Movement strategy and quality—Analysing how force is distributed and coordinated during functional tasks, including trunk and pelvic control, braking strategy and limb loading behaviour.
3. Psychological readiness—Evaluating confidence, apprehension and perceived readiness to return to high‐demand activity.

The interaction between these dimensions contributes to the identification of individualised recovery patterns and potential rehabilitation priorities. To facilitate clinical interpretation, the present framework may be illustrated through representative clinical profiles.

### Illustrative clinical profiles

Clinical interpretation within the MERItest^TM^ is based on the interaction between force production, movement control and psychological readiness, as these dimensions may recover at different rates across individuals. To illustrate this interpretive approach, two representative recovery profiles are presented below. These examples are intended to demonstrate how multidimensional findings may orient rehabilitation priorities rather than define validated clinical categories.

A first profile may reflect a deficit in rapid force expression despite preserved maximal capacity. In this situation, the athlete presents with symmetrical or near‐symmetrical isometric strength and high psychological readiness, suggesting adequate force‐generating potential and low perceived limitation. During bilateral tasks such as countermovement and drop jumps, performance may appear symmetrical, with satisfactory jump height, symmetrical vertical ground reaction force and concentric impulse. However, higher‐demand conditions—particularly unilateral tasks and rapid loading situations such as the single‐leg drop jump or single‐leg hop tasks—reveal asymmetries in rate of force development, reduced reactive strength index and impaired force absorption and reapplication. Importantly, the presence of high psychological readiness suggests that this deficit is unlikely to be primarily driven by apprehension or voluntary protective behaviour, but rather reflects a limitation in the athlete's ability to generate and control force rapidly under sport‐specific constraints, particularly in stretch‐shortening cycle conditions involving high knee extensor demand. In this profile, the limitation lies in the dynamic expression of force rather than in maximal strength itself. Rehabilitation should therefore prioritise reactive strength, eccentric control and high‐velocity, task‐specific loading.

A second profile may reflect a global deficit in capacity associated with an early and persistent protective redistribution of load. In this situation, the athlete presents with reduced isometric force production and lower psychological readiness. This is accompanied by asymmetrical force production even during bilateral tasks, with reduced vertical ground reaction force and impulse on the operated limb, indicating early unloading strategies. Notably, this protective pattern may already be present in relatively low‐demand conditions, suggesting a combined limitation in both physical capacity and load acceptance. During running, this may be further reflected by reduced knee extension moment and decreased knee flexion excursion, consistent with persistent underutilisation of the reconstructed knee. In this profile, the limitation is both structural and behavioural: insufficient force capacity combined with a tendency to redistribute load away from the operated limb. Rehabilitation should therefore focus on restoring force capacity, rebuilding confidence and progressively reintroducing mechanical loading under controlled conditions.

These profiles illustrate that similar levels of performance may reflect fundamentally different recovery states driven by distinct underlying mechanisms. In this perspective, discordant findings should not be interpreted as contradictory results to be simplified, but as clinically meaningful signals that help orient rehabilitation priorities and refine return to sport progression.

### Decision‐making and clinical application

By integrating these findings into a coherent profile, this test battery allows clinicians to triage intervention strategies, determine the appropriateness of sport‐specific reintegration and communicate more effectively across interdisciplinary teams (surgeons, sports physicians, physiotherapists, performance staff and psychologist). Rather than producing a binary recommendation, the proposed assessment informs a graduated reconditioning strategy. For example, an athlete with symmetrical CMJ force but asymmetrical propulsion during SLHD may be cleared for linear running but delayed for cutting or pivoting sports. Similarly, the presence of pelvic instability during running may prompt targeted lumbopelvic stabilisation work before advancing toward plyometric drills. Ultimately, the MERItest^TM^ enhances the clinical utility of data by embedding it within a decision‐making structure that is both interpretable and adaptable to real‐world practice.

## LIMITATIONS AND FUTURE WORK

While the MERItest^TM^ offers a clinically grounded, biomechanically rich framework for assessing recovery at six months post‐ACLR, it is not without limitations. Recognising these is essential both to contextualise its current utility and to guide its evolution.

First, the present test battery provides a high‐resolution snapshot of the athlete's neuromuscular status at a single time point. However, return to sport readiness is not binary and recovery is rarely linear. A one‐time assessment, even when multidimensional, risks misclassifying an athlete whose progress is lagging or accelerating. Future iterations should consider serial applications to map recovery trajectories, identify plateaus and better capture individual variability.

Second, all tasks are performed in a planned and anticipatory context. This limits the test's ability to capture reactive motor control, attentional load management, or adaptability under unanticipated stimuli, factors increasingly recognised as critical to non‐contact ACL injury mechanisms. While closed‐skill tasks are appropriate for structured assessments, integrating perturbation, dual‐tasking, or unanticipated decision‐making could enrich the ecological validity of the protocol.

Third, the current task battery emphasises vertical and horizontal movement directions, with no dedicated assessment of lateral or rotational task. This limits the comprehensiveness of the movement profile, particularly with regard to readiness for pivoting sports. However, this choice was deliberate, as multiplanar closed‐skill tasks such as change‐of‐direction manoeuvres may impose substantial demands that are not appropriate or safely standardised for all athletes at 6 months post‐ACLR. Future developments of the framework may therefore consider the inclusion of frontal‐ and transverse‐plane tasks at later stages of rehabilitation, when athletes can be exposed more safely to these higher‐demand movement conditions.

Fourth, the proposed assessment relies on high‐fidelity technology, including three‐dimensional motion capture, force plates and fixed‐frame dynamometry, to ensure a high level of measurement precision. While this constitutes a major strength in terms of sensitivity to subtle neuromechanical deficits, it may limit accessibility outside specialised centres. At present, the MERItest^TM^ should therefore be considered an exploratory framework designed to support both research and clinical reasoning, rather than a validated tool for immediate widespread clinical implementation. Its primary objective is to provide a high‐resolution characterisation of neuromuscular and biomechanical recovery following ACLR. In a research context, the framework may serve as a phenotyping tool to identify neuromechanical recovery profiles across individuals and to better understand the interactions between strength, movement strategy and psychological readiness. Such an approach may contribute to identifying patterns that are not detectable using conventional return to sport criteria.

From a clinical perspective, the present framework is intended to support the structured interpretation of multidimensional data and to guide individualised rehabilitation strategies. This rationale is supported by preliminary work in which only moderate correlations were observed between quadriceps strength and selected biomechanical variables during hop and jump tasks at 6 months post‐ACLR [6]. These findings suggest that strength contributes to dynamic performance, but does not fully explain how movement is mechanically expressed, thereby reinforcing the need for a multidimensional framework integrating both physical capacity and biomechanical behaviour. However, its direct impact on clinical outcomes, including return to sport decisions and reinjury risk, remains to be established. An important perspective of this work is that insights derived from high‐resolution assessments may inform the development of more accessible clinical tools and decision‐making strategies.

Future work will first aim to apply the present framework in larger single‐centre cohorts to characterise multidimensional recovery profiles and better understand recurring patterns of neuromechanical recovery after ACLR. On this exploratory basis, subsequent studies should evaluate the reliability, feasibility and prognostic value of the framework, including its relationship with return to sport trajectories and reinjury‐related outcomes. A further objective will be to identify the most clinically informative dimensions of the assessment, with the longer‐term aim of translating these insights into more accessible and clinically applicable evaluation strategies.

Finally, while the present testing framework is grounded in biomechanical theory and early internal data, it has not yet been prospectively validated. Its capacity to predict re‐injury, inform return to sport decisions, or modify rehabilitation outcomes remains theoretical. Establishing its predictive value through large‐scale longitudinal studies will be essential to confirm its utility in routine practice.

## CONCLUSION

The transition from early rehabilitation to return to sport preparation following ACLR remains a vulnerable phase in athletic recovery. Despite advancements in surgical techniques and rehabilitation protocols, re‐injury rates remain high, often due to limitations in how recovery is assessed and managed. Mid‐term evaluations too often rely on isolated metrics, symmetry thresholds, or binary clearance criteria that fail to reflect the complexity of neuromuscular restoration [26].

This multidimensional test battery was developed to address this gap. By integrating monoarticular isometric strength testing, high‐resolution three‐dimensional biomechanical analysis of dynamic tasks, and a psychological readiness questionnaire, it provides a multidimensional profile of the athlete at the 6‐month milestone. Its clinical structure is designed not to 'clear' or 'fail' athletes, but to inform individualised decision‐making, detect hidden deficits and guide reconditioning with greater precision. The protocol brings together objective data and clinical reasoning into a reproducible framework that balances scientific rigour with practical feasibility.

This review has outlined the conceptual foundations and structure of the MERItest^TM^, situating it within the current landscape of post‐ACLR evaluation strategies. While its multidimensional design marks a step forward, its true clinical value will depend on future prospective validation and efforts to increase accessibility. Nonetheless, the present testing framework illustrates what is urgently needed: structured, integrative and clinically meaningful assessment models that move beyond surface‐level criteria to support safer, more individualised return to sport trajectories.

## AUTHOR CONTRIBUTIONS

Xavier Laurent conceived the conceptual framework, developed the MERItest^TM^ model, and drafted the manuscript. Damien Dodelin contributed to the conceptual framework, biomechanical expertise, methodological input, and manuscript revision. Nicolas Bouguennec contributed to the clinical perspective, conceptual refinement, and critical revision of the manuscript. Nicolas Graveleau contributed to the clinical perspective, conceptual refinement, and critical revision of the manuscript. All authors read and approved the final manuscript.

## FUNDING INFORMATION

The authors have no funding to report.

## CONFLICTS OF INTEREST STATEMENT

The authors declare no conflicts of interest.

## ETHICS STATEMENT

Ethical approval was not required for this narrative review, as no human participants were involved and no new data were collected.

## Data Availability

No new data were generated or analysed in this study.
