# Correction to “MERItest™—A multidimensional model for functional evaluation at 6 months after ACL reconstruction”

**DOI:** 10.1002/jeo2.70958

**Published:** 2026-10-29

Laurent X, Dodelin D, Graveleau N, Bouguennec N. MERItest™—a multidimensional model for functional evaluation at 6 months after ACL reconstruction. *J Exp Orthop*. 2026;13:e70836. https://doi.org/10.1002/jeo2.70836

In the Author Contributions section, the following statement was incorrect: “Xavier Laurent conceived the conceptual framework, developed the MERItest^TM^ model, and drafted the manuscript. Damien Dodelin contributed to the conceptual framework, biomechanical expertise, methodological input, and manuscript revision. Nicolas Bouguennec contributed to the clinical perspective, conceptual refinement, and critical revision of the manuscript. Nicolas Graveleau contributed to the clinical perspective, conceptual refinement, and critical revision of the manuscript.”

This statement incorrectly attributed the conception of the conceptual framework and the development of the MERItest™ model solely to Xavier Laurent and did not accurately reflect the collaborative contributions of all authors.

The correct Author Contributions statement should have read: “All authors contributed to the conception and development of the conceptual framework and the MERItest^TM^ model. Xavier Laurent drafted the manuscript. Damien Dodelin contributed biomechanical expertise and methodological input. Nicolas Bouguennec and Nicolas Graveleau contributed to the clinical perspective and conceptual refinement. All authors critically reviewed and revised the manuscript and approved the final version.”

We apologize for this error.

